# “I was also trying to protect myself and save my life,” experiences of people living with severe mental illness and their caregivers regarding COVID-19 response in Uganda

**DOI:** 10.1017/gmh.2024.67

**Published:** 2024-05-30

**Authors:** Noeline Nakasujja, Racheal Alinaitwe, Janet Nakigudde, Andrew Turiho, Harriet Birabwa-Oketcho, Seggane Musisi

**Affiliations:** 1 Department of Psychiatry, Makerere University College of Health Sciences, Kampala, Uganda; 2 Butabika National Referral Mental Hospital, Kampala, Uganda

**Keywords:** COVID-19, experiences, people with severe mental illness, caregivers, Uganda

## Abstract

**Introduction:**

People with severe mental illness (SMI) are highly vulnerable and more affected by epidemics than the general population. They encounter limited access to care, miss out on infection prevention measures and are more prone to relapses.

**Objectives:**

This study explored the experiences of individuals with SMI and their caregivers in Uganda during the COVID-19 pandemic. Its focus was on the impact of COVID-19 and its response measures on their mental health.

**Methods:**

The study was conducted at three sites; a national referral mental hospital, a regional referral hospital and a district hospital. Participants included persons with SMI, their caregivers and mental health professionals. Data collection involved in-depth interviews, key informant interviews and focus group discussions. Phenomenological thematic analysis was employed.

**Results:**

The key themes identified encompassed challenges in accessing mental health services, disrupted routine care, the impact of lockdown measures and discrimination.

**Conclusion:**

The findings highlight the unique challenges faced by individuals with SMI and their caregivers during the COVID-19 pandemic in Uganda. There is need for interventions focusing on continued access to care, improving information dissemination and addressing the psychological impact of containment measures on people with SMI.

## Impact statement

This article focused on the experiences of people living with severe mental illness and their caregivers during the COVID-19 pandemic in a resource-limited setting in Uganda. It also looked at the use of a mobile mental health clinic in addressing the challenges of access to care by bringing mental health services closer to the communities where people with SMI reside. The findings of this research show that people with SMI are a unique and vulnerable population with unique needs that should be addressed during the response measures of a pandemic, especially in low- and middle-income countries like Uganda.

## Introduction

Evidence and experience from past epidemics show that people with severe mental illness (SMI) are a highly vulnerable group and are more negatively impacted by epidemics than the general population (Moreno et al., 2020). Higher rates of COVID-19 infection and concerns that increase susceptibility to severe illness such as comorbidity and substance abuse have been noted among those with SMI (Byrne et al., 2021). Literature shows that persons with SMI were greatly affected during the COVID-19 pandemic; a study by Muruganandam et al. (2020) in India showed that only 25% of persons with SMI were aware of the symptoms of COVID-19 and the preventive measures against infection hence 75% reported not being worried about contracting COVID-19 (Muruganandam et al., 2020). People with SMI have also been shown to have challenges in accessing care for their pre-existing mental illnesses, adhere to their medications and are more likely to relapse and have severe psychological distress (Cullen et al., 2020; Muruganandam et al., 2020; Van Rheenen et al., 2020; Neelam et al., 2021). SMI clients are a vulnerable population because they are frequently stigmatized, and left out of health education messaging and interventions for healthcare (Maling et al., 2011; Kahl and Correll, 2020; Kozloff et al., 2020). People with SMI have cognitive deficits that could impair their interpretation of educational messages given during the COVID-19 pandemic hence affecting behavior change for exposure and infection prevention (Shinn and Viron, 2020). They are often neglected by family, have few or no support networks and may engage in behaviors exposing them to infection, for example, using addictive substances, wandering on the streets when psychotic or having “I don’t care suicidal ideations” (Kozloff et al., 2020). People with SMI, including bipolar, schizophrenia and major depression have an increased COVID-19-related mortality, thus making this vulnerable population more disadvantaged (De Hert et al., 2022). Those who were acutely ill found it difficult to adhere to the COVID-19 control measures like lockdown, stay home orders, wearing masks, hand hygiene and curfew times (Shinn and Viron, 2020). The fear, anxiety and stress brought on by the pandemic may exacerbate their conditions or lead to other mental disorders such as post-traumatic stress disorder, panic anxiety and depression (Pfefferbaum and North, 2020; Shinn and Viron, 2020).

Despite increased demand for mental health services during the COVID-19 pandemic, the response measures such as restricted movement, quarantines and lockdowns affected access to mental health services (Neelam et al., 2021; Theis et al., 2021). Routine care for mental illness was tampered with as resources were diverted to mitigate the physical effects of the pandemic (Neelam et al., 2021). Mental health facilities at regional hospitals in Uganda were converted into COVID-19 treatment units further hindering access to mental health care (Mwesiga et al., 2021). Attention was focused on the emotional disturbance of infected persons, frontline health workers and the general public but the experiences, concerns and impact of COVID-19 on people with SMI went unaddressed (Neelam et al., 2021).

SMI is defined as a mental, behavioral or emotional disorder according to the DSM or ICD for more than 2 years which results in serious functional impairment (Wiersma, D. (2006). This study aimed to explore the experiences of individuals with SMI and their caregivers in Uganda during the COVID-19 pandemic. It focused on the impact of the pandemic and its response measures on their mental health, access to care and overall experiences. This article presents only the qualitative data that focused on assessing the effect of the COVID-19 pandemic and its response on people with SMI and their caregivers. We received funding from the COVID-19 Africa Rapid Grant Fund through the National Research Foundation to conduct research among people with SMI.

## Methods

This study was part of a bigger study that employed a mixed methods approach to explore the experiences of people living with SMI during the COVID-19 pandemic. In addition, the study sought ways of mitigating these challenges by employing mobile mental health clinic outreaches. The study took place at three study sites namely: Butabika National Referral Mental Hospital, Masaka Regional Referral Hospital and Mityana District Hospital. These sites were chosen because of their diversity in terms of geographical location yet having cultural similarity in Uganda’s predominantly Luganda-speaking Central region. Butabika had a mainly urban population, Masaka an up-country semi-urban population and Mityana had a rural population.

The study was conducted shortly after the second wave/lockdown of the COVID-19 pandemic in Uganda. The lock down measures included the following; only essential categories of workers were allowed to work, there were no public means of transport and private cars were allowed to move, if only they had government-provided stickers. Motorcycles were allowed to carry goods but no passengers. Public places like schools and churches were closed, and there was a ban on public gatherings. The lockdown of 2 years from March 2020 to January 2022 was one of the longest in the world. The country also had a curfew that restricted nightlife, businesses and movement beyond 7 pm (Mutono and Zotto, 2020).

The study participants were persons with SMI, their caretaker/family members and their mental health professionals who were identified at the respective hospital outpatient clinics. The inclusion criteria were based on a previous study we had conducted in these hospitals on patients with SMI (Alinaitwe et al., 2024).

Inclusion criteria focused on persons with a primary diagnosis of SMI (Schizophrenia, bipolar disorder, major depression, chronic substance dependence and epilepsy); aged 18–65 years old; receiving treatment at the study site for at least 6 months; having capacity to provide informed consent; able to communicate in Luganda or English, and able to identify their caregiver/family member. Patients with chronic physical medical illnesses, including HIV/AIDS (by self-report), and those who were inpatients at the time of recruitment were excluded. The caregiver/family member was an individual living with the person with SMI in the same household, aged 18 years or older, and participating in the provision of assistance needed in meeting the daily needs of the SMI service user. The mental health professionals were those healthcare professionals who had worked with the service users at the respective study sites for at least 6 months and with no plans to move out of the area within the next 1 year.

The research assistants (RAs) informed the service users about the study in the outpatient waiting area. Then, the clinicians administered the ICD-10 to the participants while accessing care to diagnose SMI. The RAs then assessed the capacity to consent for these service users using the University of California, San Diego, Brief Assessment of Capacity to Consent. Eligible participants were recruited consecutively till a sample size of 30 servicer users and 30 family members (1 per family) per site was achieved. Three service users and three family members from each site were purposively selected for in-depth interviews (IDI). We also similarly selected three mental health professionals for the key informant interviews (KII). We conducted two focus group discussions (FGD) per site with six to eight participants; one for service users and the other for caregivers. The FGDs were facilitated by the mental health professionals who were earlier trained in group facilitation and qualitative data collection. The IDIs and KII were conducted by RAs who were also trained in qualitative data collection.

Permission was sought from the hospital administration at each of the study sites, and meetings were held with the respective site Psychiatric Clinical Officer/nurse, Psychiatrist, study PI and RAs. During the meetings, discussions were held regarding the study procedures, study instruments and their administration and the outreach mobile mental health clinic intervention that facilitated access to care during the COVID-19 pandemic.

The discussion topic guides were formulated for the qualitative data collection with guidance of the qualitative experts on the study team. The focus was on the concerns of the SMI service users, their family caregivers and mental health professionals in relation to medication, stigma, transport, care access, lockdown, curfew, lived experiences in the pandemic, and challenges they faced as well as any other topic of their concern. The RAs selected participants for the qualitative interviews following checklists formulated by the qualitative experts. The checklists focused on both male and female representation, youth and elderly representation, those who were enthusiastic about the study, ability to speak freely in a group setting and those able to speak Luganda well. The qualitative interviews were audio recorded.

Recruited site mental health professionals, patients and their preferred family caregivers provided written informed consent after the RA had explained the study details to them including risks and benefits. Demographic information was collected from each of them before each respective qualitative interview.

The interview guides focused on access to care, perception of health messaging and knowledge information given to the patients regarding the COVID-19 pandemic, stigma and the effect of the pandemic containment measures (government response) on patients and their families in relation to the SMI (e.g., relapses, hospitalizations, substance abuse and traumatic experiences).

For data analysis, qualitative audios from the IDIs, KIIs and FGDs were transcribed and translated into English. Data analysis and management was done by experienced qualitative researchers on the team employing phenomenological thematic analysis (Miles and Huberman, 1994) using Atlas-ti qualitative analysis software. Draft codes were developed and discussed with the rest of the team. Similar codes were grouped under themes, and the identified themes and sub-themes were then checked and refined by the qualitative experts (JN & AT) on the study team. The analysis employed an inductive approach to provide new insights and a richer understanding of the data. Verbatim quotes from the data are reported in the results.

## Results

### Sociodemographic characteristics of the respondents

Twenty-three (23, 76.7%) SMI participants in this study had attained primary or secondary education, almost half (43.5%) were not in employment and 56.5%, were females.

The thematic qualitative analysis revealed themes elaborated below revolving about the experiences, beliefs and attitudes of SMI clients and their families during the COVID-19 pandemic and its response measures, the effect of COVID-19 pandemic response measures to access to care and the effect of the pandemic and the response measures on mental health of people with SMI.

### Access to healthcare

The COVID-19 pandemic affected people with SMI and their caregivers through a curtailing of access to health care, thus increasing the risk of relapse in addition to resulting into other negative social and economic effects. With a varied compliance by both service users and caregivers to the COVID-19 response, infections and deaths were reported in the communities around them.

#### Support

Perceived support including clear explanations regarding COVID-19 infection from caregivers and clinicians enhanced compliance with the COVID-19 Standard Operating Procedures (SOPs) in the service user population. It appeared that clear communication and the simplicity and clarity of messages were factors that facilitated the observance of COVID-19 SOPs. A service user participant indicated that the support from his family enabled him to protect himself as can be seen from this quotation:

*“My family urged me so much not to go to crowded places; they told me, if I moved, I wouldn’t know what I would pick out there. And since they were supportive, I did not find any difficulty obeying… I had no problem because I was also trying to protect myself and save my life”***(IDI, service user at Butabika).**

#### Health education messaging

Additionally, messages that were disseminated through health workers to service user participants were strongly observed by the participants. This is probably because health workers were held in high regard and esteem. A caregiver of a service user alluded to this idea in the quotation below:

*“My patient strongly respects health workers’ words. Like the Covid vaccination; he was willing to do it. He is always reluctant about modern medications but he responded”* **(FGD, Caregiver at Masaka).**

A similar sentiment from another caregiver still indicated that when clinicians communicated to the service user participants, they listened and took their advice as is indicated below:

*“… Sitting and listening to a clinician directly is more effective. When a clinician advises them (patients), they listen to what they are told…”* **(FGD, Caregivers at Masaka).**

#### Adherence to SOPs

The significant mortality and morbidity due to COVID-19 was a scare to many and it was perhaps one of the greatest enablers of compliance with COVID-19 SOPs. Many people disregarded the COVID-19 SOPs initially until they witnessed the loss of lives. Regardless of how actively severely ill SMI service users were, many of them were still aware of the SOPs and they endeavored to take the necessary steps to protect themselves. Here are some statements of caregivers and service users:

*“At first people didn’t take it serious but when people started dying, we strictly followed the preventive measures”* **(FGD, service users at Masaka).**

A response by one of the caregivers in an FGD alluded to the idea that hearing about numerous deaths due to Covid-19 prompted people to start taking protective measures seriously, as is illustrated below in a quotation:

*“In the community when people heard of the high number of deaths, they strictly followed instructions. They also limited visiting each other… People feared and followed SOPs when they heard that so many people in the city were dying”* **(FGD, Caregivers at Masaka).**

There is an indication from the discussions that even when SMI service users were actively ill, many still took the necessary measures to protect themselves from Covid-19 infection. One caregiver noted:

*“Mine (SMI patient) used to care so much… she would never forget the mask even during church service; that is at the time when we were allowed to go to churches. She would ask for the mask all the time yet that was the time when she was not mentally stable…”* **(FGD, Caregivers at Masaka).**

Notwithstanding the fact that the high mortality and morbidity due to COVID-19 caused a scare and was an enabler for individuals with SMI to follow SOPs, there were severe cases of SMI that did not follow SOPs probably because the illness symptoms interfered with concentration and understanding of the need to protect one from catching COVID-19. It was noted that patients with severe symptoms had challenges in comprehending and complying with the SOPs. Some patients were reportedly too ill to even bathe on their own, let alone comprehend the basic instructions regarding COVID-19 prevention like washing and or sanitizing hands and wearing of face masks. One of the affected participants indicated that:

*”We were given information about the COVID-19; but for mentally ill patients, our brains couldn’t understand it. Even when we did, we would understand very little"* **(FGD, Service user at Butabika).**

### Perceptions regarding the pandemic

#### Misinformation and disinformation

The second major theme was the perceptions of SMI service users and their caregivers regarding the COVID-19 pandemic. Some people’s perceptions interfered with compliance to the COVID-19 pandemic SOPs. Some did not believe that COVID-19 actually existed due to misinformation and disinformation. Some SMI service users as well as their caregivers were reportedly reluctant to wear masks unless they were forced to. They reported difficulty breathing and a lot of discomfort while wearing a mask. They said that:

*“Washing hands was easy but putting on his mask was a challenge. He (patient) claimed that the mask would interfere with his breathing. We were not used to moving with our mouths covered”* **(FGD, Caregivers at Butabika).**

Also, another participant in an IDI elucidated that:

*“In school, we were taught that the air you breathe in (oxygen) is different from the air that we breathe out (carbon dioxide). Yet with a mask the same air that you breathe out is the same air that you breathe in. So, I thought that may be, we would avoid COVID-19 and get other diseases”* **(IDI, Service user at Butabika).**

#### Rumors, beliefs and misconceptions

Compliance was further complicated by beliefs and misconceptions based on rumors, especially with regard to the reality and cause of COVID-19 as well as vaccination against COVID-19. It was noted that some people invoked witchcraft when they or a close person contracted the disease. These were therefore least likely to observe the COVID-19 SOPs. Some people had misconceptions about the adverse effects of the vaccine.

*”They (patients) refused to get vaccinated claiming they would die after two weeks (from vaccination) and that some won’t be able to give birth…… We were told that if you have a mental problem, you are not supposed to get vaccinated all the doses. People in the community scared us that the vaccination was meant to kill us”* **(FGD, Service users at Masaka).**

#### Political atmosphere

At the time of conduction of the study, there were up-coming presidential elections in the country. Many people believed that COVID-19 was a political tool used by the incumbent government to limit the political activity of the opposition and therefore tended to disregard the SOPs during the first wave. The political environment at the time impacted on the use of COVID-19 SOPs in this community, as is alluded to in the following quotation:

*“In my community, people took long to believe (COVID-19) and most of them related it to politics; that was in the first wave. So most of them only came to believe it in the second wave… At the end of the day many people died”* **(FGD, Caregivers at Masaka.**

#### Fears and worries

Study participants reported experiencing fear of contracting and spreading COVID-19. They were thus forced to take extreme precaution measures. This is elaborated by a clinician in the following quote:

*“… We would leave for home after bathing and the moment we would get home we would go straight to the bathroom and even the shoes would be washed. We developed a lot of fear”* **(KII, Clinician at Butabika).**

Some service users also experienced fear of their mental health deteriorating with relapse of symptoms due to the stress of restrictions in movement as echoed in the following quote:

*“Yes, I was so worried about not getting medication when transport was closed because when I do not get medicine, I won’t be able to sleep and if I don’t sleep the (mental) disease would easily come back”* **(IDI, Service user at Butabika).**

Many people were dying due to COVID-19 and other causes, but the burial of the dead became distressful for fear that one could contract COVID-19 in the burial crowds. Participants also reported psychological distress accruing from the inability to take care of their affected dear ones who were in isolation and also the sirens from ambulances that were believed to be transporting COVID-19 patients to hospital or dead victims for burial:

*“The way Covid infected patients were taken care of in isolation was distressing. They could have recovered but for failure to be in touch with others… This makes me believe that some may have died because of the isolation they were in. I saw many people that took care of their own patients in homecare and they recovered”* **(FGD, Caregiver at Butabika).**

### Caring for people with SMI

Another major theme that came out of the conversations with the study participants was the way in which the COVID-19 pandemic impacted on the care of people with SMI.

#### Movement and transport restrictions

There was restricted movement to health centers especially in curfew periods. Some service users moved long distances to get to hospital to attend for their clinic reviews. Banning of public transport and enforcement of curfew hours during the lockdown curtailed movement to hospitals for reviews and medicine refills. Below are some observational statements from clinicians and caregivers:

*“The biggest challenge we got is when we were restricted from carrying passengers on a motorcycle… We only have one hospital that treats mental illness… there wasn’t any means of transport. And you couldn’t move on foot due to the long distance. People had to resort to bicycles to ride for about 30 miles”* **(FGD, Caregivers at Masaka).**

Also

*“Curfew affected us; those were few hours for one to do what they had to do. Even the security forces meeting you and they just beat you up. It was too hard to find vehicles. Some were arrested yet at Police they did not observe SOPs; prisoners were at a high risk of exposure to COVID-19. Us who would work would be forced to leave early because of the tension from security officers”* **(KII, Clinician at Butabika).**

Some clinicians, however, lived in staff quarters but others lived far off. Most clinicians reported coming across some security personnel who respected health workers and spared them during curfew. This enabled them to continue providing health services in the hospitals.

*“I did not have issues with curfew personally since I was staying in the staff quarters… if you are a medical staff and wanted to go somewhere, we used our identity cards whenever we got problems. That was on my side; I don’t know what the other staff experienced”* **(KII, clinician at Masaka).**

#### Law enforcement

The COVID-19 period came in with stringent SOPs on movement with dire consequences enforced by police when these were not adhered to. Sometimes, the victims felt that too much force was used by the police, and sometimes, to extricate themselves from this trouble, they would bribe the police to avoid arrest. A caregiver who fell prey to this had this to say;

*“The force which the police was using to handle those arrested was too much. It would have been better if they had sensitized people other than just arresting them, then again take away the little money they had. That was a lot of torture.” Like the day I moved out when someone offered me a job… The policeman saw me and waited till it was past curfew time; he put me in jail and I ended up paying a UGX 50,000 bribe. (US$ 15).”* **(FGD, Caregivers in Masaka).**

The occurrence of the COVID-19 pandemic was a new experience for everyone in the country and was the very first time they were experiencing a pandemic of such magnitude.

#### Limited SMI admissions

In an effort to accommodate the vast numbers of patients affected by COVID-19, the Ministry of Health displaced mental health spaces in hospitals and designated them to COVID-19 management. The reassigned mental health spaces were inadequate, and the observance of SOPs was a challenge for service delivery. These units were also understaffed; hence, there was prolonged waiting and an overstretched staff. Also prior to COVID-19, admissions in the mental health units were available. With the advent of COVID-19, there were limited SMI admissions. This was a disadvantage to the SMI patients who would have to travel back to their homes in quite often agitated/disturbed mental states. These concerns are reflected in the following quotes of clinicians and patients.

*“Before COVID-19 came, we had our unit but all of a sudden, we were told to vacate the place; it was on short notice… They told us we were going to work at OPD. After like a week… medical clinicians up there were also complaining that we occupied the place yet they wanted to use it; we had to again leave… but patients were coming… until they took us miles away to Kyabakuza (Health Centre II). It’s where we settled till now”* **(KII, Clinician at Masaka).**

*“Those days (prior to COVID-19) you would get admitted and they would treat you for some time until you got fine, unlike in the COVID-19 lockdown where patients weren’t admitted”* **(KII, Clinician at Masaka).(FGD, service user at Masaka).**

#### Shortage of medical staff

Second due to the excess staffing demands that the pandemic required, a good number of clinicians were sourced from the mental health arena leaving fewer staff to work on patients with mental health issues.

*“The psychiatric clinicians were fewer compared to the times before COVID-19. When the clinicians are few you take long to leave and start feeling hungry. Yet if they are many you spend little time in the line and go back home”* **(IDI, Service user at Butabika).**

### Accessing mental health care

#### Medicine stock-outs and increased prices

The challenge of accessing mental health care was compounded by psychiatric medicine stock-outs in public hospitals and private pharmacies. Mental health clinicians testified that facilities ran out of medicines and people had to buy from private sources to avoid illness relapses. The cost of medicine shot up during the pandemic period, rendering it largely inaccessible, as highlighted in the following narrative by a caregiver.

*“We got to a point where we did not have medicines in hospitals… even in the pharmacies…. One time I was even sent to Kampala and I bought the medicine very expensively; … ”* **(FGD, Caregivers at Masaka).**

In the rural areas, many of the service users seen at the facility used to be supported by their relatives who live in the cities in terms of buying medicines. After closure of many business and work places, these resorted to selling personal property to sustain themselves. As also attested by a caregiver in Masaka (below), high poverty levels attributable to the lockdown indeed impacted the quality of care that caregivers extended to their patients.

#### Caregiver support

The amount and quality of caregiver support was impacted upon by COVID-19 as is indicated in the following quotation:

*“We also had a challenge of finances… As an individual you may desire to do a lot like buying your patient a drink but you wouldn’t have money as you may be required to buy some medicines from a downtown pharmacy because it was not enough in the hospital… At the end of the day the patient misses out on some of the doses which should not happen…”* **FGD, Caregiver at Masaka).**

The quality of care that people with SMI received from their caregivers was also impacted upon. When the caregiver was sick, this affected the accessibility of not only medication but also food. Another caregiver said:

*“I faced a challenge because we were two patients. I was sick and my patient too was sick and we were only the two of us in the house… We moved to the village because I could not cook for him, but I would not also take care of myself since I was weak…I fell sick to the point of admission… We reached a point where we would even run out of drugs…”* **(FGD, Caregivers at Butabika).**

Some service users, however, had family members who were still able to support them materially and financially during the lockdown. One patient said:

*”It did not affect me badly because my family members were supportive unlike my tenants who left without paying my rent. But my family was so supportive, they would send me basic needs like soap, money etc."* **(IDI, Service user at Butabika Hospital).**

#### Hospitalizations of SMI service users

COVID-19 restrictions variously affected the admitted patients as well as their caregivers. When patients who got admitted at Butabika hospital tested positive for COVID-19, one of the challenges they faced was observance of SOPs to prevent further spread of the disease. Moreover, there was inadequacy of space for isolation of suspected and confirmed cases. Clinicians noted:

*“We did not have space and we were always around them (COVID-positive patients); though we would wear masks and protect ourselves, we still got worried but mainly the patients because they feared that these are the people they stayed with and spent most time with and they are positive….By the time we got these patients, the hospital had not created isolation space for them…we did not even have a ward that would accommodate all of them for treatment…”* **(KII, Clinician at Butabika).**

Patient numbers on the psychiatric wards increased as more SMI service users were brought from other (closed) health facilities, yet transfer of patients between wards and even discharges were not allowed at some point. This led to overcrowding on the wards and fear among the resident patients that they would be infected by newcomers as well as those who had not yet shown symptoms of the disease. Admitted patients were also denied receiving visitors, which deprived them of supplies from their family members and also worried the family members since they could not ascertain the condition of their admitted relatives.

*“They (relatives) were affected because most times they wanted to visit their admitted patients; so they would request (call) them to come out and we would insist that it would not be possible. The caretakers got so worried when they noticed that the numbers (of COVID cases) were big”* **(KII, Clinician at Butabika).**

#### Medication noncompliance and relapses

Many people were not able to work to earn money during the pandemic lockdown. This escalated poverty levels, yet transport fares were hiked. This affected access to health care in the sense that they could not afford the high cost of commodities and transporting service users to hospital. This reportedly led to increased rate of noncompliance to medication and consequent relapses as can be seen below.

*“The rate (of relapse) was really high because of poverty; people did not have food, and they couldn’t buy drugs”* **(KII, Clinician at Masaka).**

*“We spent almost a year without him getting medication. Of course, he got a relapse and worsened. He became aggressive; he would beat and fight us”* **(FGD Caregiver, Butabika).**

*“Since my sister had gone off medication, we were worried about her getting a relapse because she wasn’t able to move from home to hospital. Even if one had a bicycle you wouldn’t be allowed to ride it to the hospital”* **(FGD, Caregiver at Butabika).**

#### Home, school and social disruptions

Lockdown occasioned various forms of social and economic losses to affected households. Some participants missed their hospital visits and the interactions with friends, family members and relatives on these visits. Domestic quarrels reportedly increased when family members were made to stay at home all the time. Service users relapsed due to disruption of medical care, which rendered them unable to perform certain roles, including attendance of important family functions. For example, a service user participating in an FGD in Masaka testified that his daughter wedded when he had relapsed and he had no recollection of what took place at the time. There was also a loss of spousal support in the form of companionship and contribution to family economic activities like farming.

*“My wife got to a point where she couldn’t do most of the things; for example she used work in the garden and we would share responsibilities. But now I am the only one who is attending to everything… when she got off medication it affected me so much… I always moved out with my wife which I could not do any more…”***(FGD, Caregivers at Masaka).**

People were not able to leave home and go to work where they were supposed to earn a living due to the lockdown. At some point, motorcycle taxis (*boda-bodas)* were only allowed to carry luggage and no passengers. Consequently, household resources including food got depleted. Disruption of livelihoods stressed both caregivers and servicer users as reflected in the quotes below.

*“Feeding was hard since we were all not working. I was at University at the same time working but during the COVID-19 pandemic, we were not able to work so we could only feed on whatever little we were able to get”* **(FGD, Caregivers at Butabika Hospital).**

*“Whenever mummy (service user) didn’t have money, she would be so tough, that was my biggest challenge… she would always be rude, she would over sleep. I remember there is a time she blacked out and she took three days without waking up then she would fall sick again; all the time”* **(FGD, Caregiver at Masaka Hospital).**

*“We are not financially stable and it will take us a lot of time to get back on our feet. We are literally starting from scratch. You start up a business and it simply does not thrive”* **(FGD, Caregivers at Butabika).**

Social life was variously affected. All participants reported being deprived of spiritual nourishment and social networking opportunities for lengthy durations since they were not allowed to congregate in places of worship. This affected many categories of people including the clinicians. They said:

*“My biggest challenge was not being able to go to church for prayers, because these were closed. I was kept away from the presence of God”* **(IDI, service user in Masaka).**

*“We were refused to attend mosques; I am a Muslim, it was really affecting me in a way that people were not allowed to go to places of worship., they were bored wishing they would be allowed to go back and pray” (KII, Clinician at Masaka).*

#### The cost of suffering and losing loved ones

Participants reported personally suffering from COVID-19, having dear ones infected and losing dear ones to COVID-19. A participant in the FGD of service users at Butabika narrated how he found it terrifying to look after his friend who got COVID-19 and recovered. A family member from Masaka who developed the symptoms reportedly did not get tested and instead got medicine from a pharmacy for fear of being quarantined since they were not sure what was happening to those already quarantined. The fear of contracting the virus also affected the clinicians as well as caregivers as cited below.

*“When we saw how people were dying in other countries on TV, yet those people were considered powerful (developed) countries, we thought we were just going to perish like grasshoppers but God is faithful, it did not happen”* **(IDI, Caregiver in Masaka).**

*“The experience I had was the fear of contracting the disease, because our servicer users come touching anywhere, without washing, without masks and coughing anyhow, so there were high chances of contracting the disease…”* **(KII, Clinician at Masaka).**

Other than the physical and psychological pain occasioned by COVID-19 infection, the cost of treatment was reportedly high, yet most people were not working. A service user participating in an FGD at Masaka reported that he contracted COVID-19 and spent a lot of money to get treated. A member of such an affected family in Masaka reported that a dose of treatment cost at least UGX 60,000 (US$ 17). A service user in the same FGD lost the person who used to meet his medical bills, leaving him largely unable to get treatment. A caregiver in the FGD at Butabika lost an uncle and an aunt in the same year. Another participant in the same FGD contracted COVID-19 and also lost a brother and a relative.

## Discussion

This study has highlighted the lived experiences of service users with SMI, their caregivers and clinicians during the COVID-19 pandemic in Uganda. We captured experiences related to compliance with COVID-19 measures and SOPs among people with SMI as well as various psychological and socioeconomic impacts related to the COVID-19 pandemic. These findings highlight the experienced disruptions to access to mental health care among the SMI service users and call for adaptations and innovations that are unique in our low resource settings.

In the context of the pandemic, infection control is an immediate need and the need to comply with COVID-19 response measures is of crucial importance in both community and hospital settings. Our participants alluded to the wide and appropriate dissemination of information on COVID-19. However, several factors influenced the participants’ compliance with the COVID-19 response measures. These factors included poor access to SOP materials, severity of the mental illnesses, awareness of deaths due to COVID-19 and hence widespread fear and anxiety, negative attitudes and misinformation/disinformation toward COVID-19. The participants felt that information given by their clinicians directly through regular service users or caregiver group meetings was more beneficial to and believable by people with SMI. We came across no studies looking at SMI service users’ compliance with COVID-19 response measures at the community level. Our findings point to the need to emphasize targeted messages for people with SMI.

SMI service users’ compliance with SOPS was particularly difficult in the inpatient settings. Many studies have reported about the complexity of infection control within mental health settings (Bojdani et al., 2020; Kahl and Correll, 2020; Gillard et al., 2021; Johnson et al., 2021), and this study agrees with these findings. There was inadequate space for isolation in an already crowded setting of SMI service users who were unable to practice effective control measures due to the severe nature of their mental illnesses. This was a concern for both clinicians who were not feeling safe in the hospital setting as well as the caregivers who were not allowed to visit them. This finding echoes the recommendation by Xiang et al. (2020) to institute specific measures for mental health units during pandemics (25).

The need for uninterrupted access to mental health and support services for persons with SMI has been emphasized by WHO (2020). Our findings are similar to other studies (Sheridan Rains et al., 2021), which indicate that the pandemic led to restrictions on access to mental health care through the lockdown measures, the curfews and restricted transport measures, all of which affected service users’, caregivers’ and staff movements amidst the pandemic. In Uganda, this was made worse by the displacement of mental health services from their usual wards in the regional referral hospitals to other more isolated areas (Mwesiga et al., 2021).

Medication stock outs and higher prices as a result of the lockdown led to service users missing their medications, thereby causing relapses of their illnesses. This calls for adaptations and innovations such as new crisis services, extended services and community services that offer practical help such as drug deliveries for service users, use of remote technologies and use of informal support mechanisms, as has been recommended by some studies (Tromans et al., 2020; Johnson et al., 2021). Some participants suggested that the government should stock psychiatric drugs to lower healthcare units (Health Centers) where mental health workers could use telephone calls to reach out to the persons with SMI to call them to pick up their medications. There was minimal mention of sophisticated remote technologies such as video conferencing and the use of smart apps in this study, unlike other studies (Honey et al., 2021; Johnson et al., 2021). This is understandable given that our study population was largely rural, peasantry or unemployed and thus lacked access to the technology. This calls for innovative ideas and community mental health services, such as mobile mental health clinics, which are recommended and are being investigated in this study (Mwesiga et al., 2021).

Previous studies have also found that pandemic lockdown measures often led to the breakdown of social, economic and family safeguards. This resulted in domestic conflicts, aggression and violence to which persons with SMI were both victims and perpetrators (Sheridan Rains et al., 2021). Our participants reported that school closures led to children and youth being redundant, dropping out of school, using substances and having early pregnancies. Other studies have reported similar findings (Hoffman and Miller, 2020; Lee, 2020; Chaabane et al., 2021). These findings have important implications for future pandemic preparedness in schools and communities.

Service users in this study also experienced the loneliness and isolation as a result of the pandemic lockdowns. In fact, one service user reported “feeling like a prisoner.” This effect of the pandemic has been reported globally in all population groups (Musisi et al., 2021). This finding was not widely reported in the Ugandan setting probably because Ugandans live in large extended families. Nevertheless, this finding in persons with SMI who already have restricted social networks calls for appropriate measures to address it for this group of people.

### Limitations

This study was limited to people with SMI seeking care from tertiary and secondary mental health services. However, many people with other mental health difficulties also come into contact with health services, including primary care services, drug shops and alternative/complementary healers (Abbo et al., 2009).

This study had some notable strengths. It reports on the experiences of the COVID-19 pandemic on persons with SMI from their perspectives and the perspectives of both the formal and informal caregivers from a variety of geographical locations in the Ugandan setting. Conduction of the study was 4 months after the second COVID-19 pandemic lockdown in Uganda. Therefore, the captured information was still very fresh in terms of the experiences the respondent had gone through thereby limiting the chances of information bias. As far as we know, this was the first study of its kind in Africa regarding the experiences of persons with SMI during the COVID-19 pandemic.

## Conclusions and implications

Individuals with SMI and their caregivers faced extra challenges in accessing care services, the barriers and enablers of compliance to SOPs as well as the psychological and social impact of the COVID-19 pandemic and its response measures on the people with SMI. This study is one of the very few studies done in Africa to further emphasize that the COVID-19 pandemic exacerbated the difficulties faced by persons with SMI to access care. This work offers researchers, clinicians and policymaker direction for mental health service development in the face of an emerging epidemic. It gives direction for opportunities for new ways of working that are appropriate for low-resourced settings, such as the use of mobile mental health clinics to enhance access to care.

## Data Availability

The authors will ensure that the study dataset is available for sharing on request following the publication of this article.
