# Supplementary material for: “I was also trying to protect myself and save my life,” experiences of people living with severe mental illness and their caregivers regarding COVID-19 response in Uganda
Source: Glob Ment Health (Camb). 2024 May 30;11:e72. doi: 10.1017/gmh.2024.67 (PMC11383974; doi:10.1017/gmh.2024.67)
Supplement: Peer review history

# Editor report: Recommendation: “I was also trying to protect myself and save my life,” experiences of people living with severe mental illness and their caregivers regarding COVID-19 response in Uganda — R0/PR2

**DOI:** 10.1017/gmh.2024.67.pr2

**Affiliations:** University of Zimbabwe Faculty of Medicine, Zimbabwe

No accompanying comment.

# Editor report: Decision: “I was also trying to protect myself and save my life,” experiences of people living with severe mental illness and their caregivers regarding COVID-19 response in Uganda — R0/PR3

**DOI:** 10.1017/gmh.2024.67.pr3

**Affiliations:** London School of Hygiene & Tropical Medicine, United Kingdom of Great Britain and Northern Ireland

No accompanying comment.

# Editor report: Recommendation: “I was also trying to protect myself and save my life,” experiences of people living with severe mental illness and their caregivers regarding COVID-19 response in Uganda — R1/PR5

**DOI:** 10.1017/gmh.2024.67.pr5

**Affiliations:** University of Zimbabwe Faculty of Medicine, Zimbabwe

No accompanying comment.

# Editor report: Decision: “I was also trying to protect myself and save my life,” experiences of people living with severe mental illness and their caregivers regarding COVID-19 response in Uganda — R1/PR6

**DOI:** 10.1017/gmh.2024.67.pr6

**Affiliations:** London School of Hygiene & Tropical Medicine, United Kingdom of Great Britain and Northern Ireland

No accompanying comment.
